# Effectiveness of Motivational Interviewing in improving lipid level in patients with dyslipidemia assisted by general practitioners: Dislip-EM study protocol

**DOI:** 10.1186/1471-2296-12-125

**Published:** 2011-11-05

**Authors:** Luis A Pérula, Josep M Bosch, Julia Bóveda, Manuel Campiñez, Nieves Barragán, Juan C Arboniés, Jose A Prados, Enrique Martín, Remedios Martín, Josep Massons, Margarita Criado, Roger Ruiz, José A Fernández, Francisco Buitrago, Inmaculada Olaya, Modesto Pérez, Joaquin Ruiz

**Affiliations:** 1 Unidad Docente de Medicina Familiar y Comunitaria de Córdoba, Instituto Maimónides de Investigación Biomédica de Córdoba (IMIBIC)/Hospital Universitario Reina Sofía/Universidad de Córdoba, Córdoba, Spain; 2 Área Básica de Salud Encants (Maragall), Institut Catala de la Salut (ICS), Barcelona, Spain; 3 EAP Colmeiro, Servicio Gallego de Salud, La Coruña, Spain; 4 CAP Vallcarca, CatSalut, Barcelona, Spain; 5 Servicio vasco de salud-Osakidetza, Centro de Salud de Beraun, Errenteria, Spain; 6 Centro de Salud Lucano, Servicio Andaluz de Salud, Córdoba, Spain; 7 Centro de Salud Fuensanta, Instituto Maimónides de Investigación Biomédica de Córdoba (IMIBIC)/Hospital Universitario Reina Sofía/Universidad de Córdoba, Córdoba, Spain; 8 ABS Mataró 7, Institut Catala de la Salut (ICS), Barcelona, Spain; 9 Centro de Salud Villarrubia-Azahara, Instituto Maimónides de Investigación Biomédica de Córdoba (IMIBIC)/Hospital Universitario Reina Sofía/Universidad de Córdoba, Córdoba, Spain; 10 Centro de Salud "La Paz", Servicio Extremeño de Salud, Madrid, Spain; 11 Distrito Sanitario Córdoba-centro, Servicio Andaluz de Salud, Córdoba, Spain; 12 Hospital Regional Universitario Reina Sofía, Servicio Andaluz de Salud, Córdoba, Spain

## Abstract

**Background:**

The non-pharmacological approach to cholesterol control in patients with hyperlipidemia is based on the promotion of a healthy diet and physical activity. Thus, to help patients change their habits, it is essential to identify the most effective approach. Many efforts have been devoted to explain changes in or adherence to specific health behaviors. Such efforts have resulted in the development of theories that have been applied in prevention campaigns, and that include brief advice and counseling services. Within this context, Motivational Interviewing has proven to be effective in changing health behaviors in specific cases. However, more robust evidence is needed on the effectiveness of Motivational Interviewing in treating chronic pathologies -such as dyslipidemia- in patients assisted by general practitioners. This article describes a protocol to assess the effectiveness of MI as compared with general practice (brief advice), with the aim of improving lipid level control in patients with dyslipidemia assisted by a general practitioner.

**Methods/Design:**

An open, two-arm parallel, multicentre, cluster, controlled, randomized, clinical trial will be performed. A total of 48-50 general practitioners from 35 public primary care centers in Spain will be randomized and will recruit 436 patients with dyslipidemia. They will perform an intervention based either on Motivational Interviewing or on the usual brief advice. After an initial assessment, follow-ups will be performed at 2, 4, 8 and 12 months. Primary outcomes are lipid levels (total cholesterol, HDL cholesterol, LDL cholesterol, triglycerides) and cardiovascular risk. The study will assess the degree of dietary and physical activity improvement, weight loss in overweight patients, and adherence to treatment guidelines.

**Discussion:**

Motivational interview skills constitute the primary strategies GPs use to treat their patients. Having economical, simple, effective and applicable techniques is essential for primary care professionals to help their patients change their lifestyle and improve their health. This study will provide scientific evidence on the effectiveness of Motivational interviewing, and will be performed under strict control over the data collected, ensuring the maintenance of therapeutic integrity.

**Trials Registration:**

ClinicalTrials.gov (NCT01282190).

## Background

There is enough scientific evidence on the causal relation between increased plasma cholesterol levels and the incidence of cardiovascular events. Similarly, such risk is reduced when cholesterol levels decrease [1]. Arteriosclerosis is an inflammatory process triggered by a number of cardiovascular risk factors (CRFs). The risk attributable to any blood cholesterol level also depends heavily on the coexistence of other CRFs [2]. Therefore, the cardiovascular event risk in patients with dyslipidemia is directly related to the total cardiovascular risk, rather than to their plasma lipid profile [3].

From the perspective of primary care (PC) the role of general practitioners -GPs- is essential in the prevention of dyslipidemia, both in terms of detection and of the therapeutic approach employed. In this sense, we know that healthy lifestyle recommendations are based on more robust scientific evidence (grade A and B recommendations) than the pharmacological treatment itself, when prescribed (grade D recommendation) [4]. There are previous successful interventions for smoking and alcohol cessation and for the promotion of healthy habits [5]. However, the impact of such interventions was from low to moderate, and most of the measures are recommended for their effectiveness in reducing morbidity and mortality, rather than for the fact that there is strong evidence that PC interventions help change health behaviors [6].

Consequently, it is essential to identify the most effective strategy that GPs could use to help their patients change habits associated to cardiovascular health. Many efforts have been devoted to develop theories that explain changes or adherence to specific health behaviors. The objective is to make such theories operative on the fourth stage of Miller's pyramid, with a reasonable cost-benefit balance. To date, many of these theories have resulted in prevention activities including brief advice and counseling [7]. Within this context, Motivational Interviewing -MI- has gained in popularity during the recent years. It have proven to be effective in helping patients with specific diseases and under specific conditions change health behaviors [8]. MI was initially used to help people approach their ambivalences and change their behavior patterns [9]. MI was a transtheoretical model derived from the Client-Centered Therapy, which combined an empathetic and understanding style of counseling [10]. Simultaneously, it is a directive method for resolving ambivalence in the direction of change. MI has evolved into "a clinical style aimed at eliciting patients own motivations for making changes in behaviors in the interests of their health" [11]. In its different applications, MI has proven to be more effective than other models as the classic informative, as effective as Behavioral Cognitive Therapy with less cost in time, and it is even more effective than some pharmacological therapies in specific cases [12,13]."

As a result, the need has arisen for valid and reliable instruments allowing us to assess to what extent GPs use MI. In this sense, scant scientific literature is available, and only two instruments are worth of mention: an instrument that is based on an orthodox theoretical approach to MI: the Motivational Interviewing Skills Code: MISC [14], and its synthesized version -more applicable-, the Motivational Interviewing Treatment Integrity or MITI [15].

The second instrument is known as the Behavior Change Counseling Index, and it is based on Behavior Change Counseling [16]. Both have been proved to be effective and reliable for assessing MI skills. However, to date, few studies performed by health professionals are based on such instruments [17]. In this context, this project suggests the development and validation of a MI skill assessment method -as an essential and complementary element- more appropriately adapted to our environment: the Motivational Interviewing Assessment Scale (EVEM in Spanish).

Finally, two general conclusions can be drawn from the data available on general practitioners' professional training [18,19]: 1) Although physicians devote many hours to their training, such training does not seem to benefit patients' health; 2) The type of education which better improves patients' health is interactive training (joint work tutor-learner) and mixed training (lecture with interaction). We also know that training in clinical Interviewing is inadequate in official health professional training programs in Spain. Thus, minimum standards for clinical Interviewing are not provided in health professional training programs and the courses including it are voluntary, unregulated and are generally performed in post grade programs, as if this professional competence was not important enough or had no clinical impact at all. In this paper, an innovative, standardized MI training program will be implemented by following the models already established in scientific literature [20] -and currently included in the MINT network (Motivational Interviewing Network of trainers). We will follow the eight stages described by Moyers and Miller [21]; similarly, economical training technologies -as e-learning- will be used and educational feedback will be provided to improve clinical skills.

### Research Objectives

The primary objective of this study is to verify the effectiveness of a multi-factorial intervention based on MI and performed by GPs specifically trained to improve control over lipid levels in patients with dyslipidemia, in contrast with the usual brief advice.

The specific objective of this study is assessing whether after a 12-month follow-up, a multi-factorial intervention based on MI achieves:

• To improve lipid levels in patients;

• To reduce cardiovascular risk;

• To improve patients' diet (adherence to the Mediterranean diet and reduction of saturated fat intake);

• To increase physical activity;

• To reduce body weight in patients with overweight or obesity;

• To improve adherence to prescribed hypolipidemic drugs.

Our complementary objectives are:

• To check the effect of the MI training program on the participating GPs allocated to the experimental group.

• To validate a measuring instrument specifically designed for assessing the use of MI (EVEM scale) among GPs.

## Methods/Design

### Study Design

**Figure 1:**
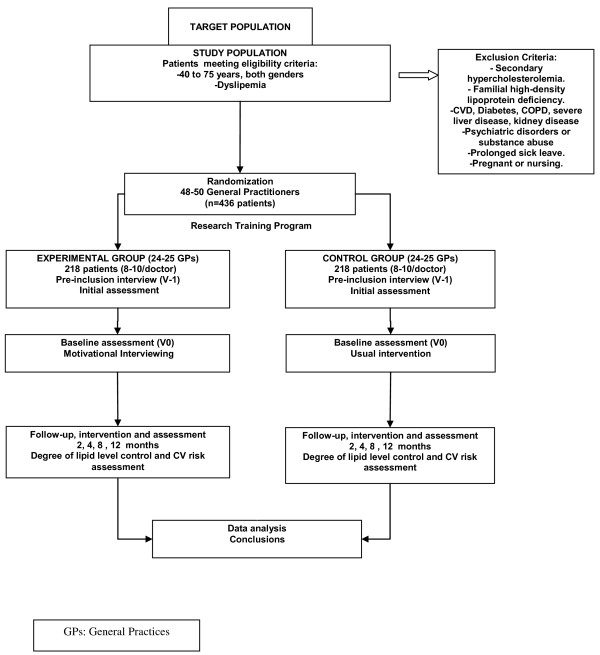
**Scheme of the Dislip-EM study design**.

It is an open, parallel, multicentre, cluster, controlled, randomized clinical trial. Two groups will be monitored for a 12-month period. Figure 1 shows a scheme of the study design.

The intervention will be based on the implementation of MI during contacts with patients, instead of the usual brief advice method. The aim is to check whether this approach is more effective in promoting cardioprotective behaviors in patients (heart protective diet, physical activity, weight loss), and in improving lipid level control, thus reducing cardiovascular risk.

### Sample Size

Basing on the results and on preliminary estimates [22-24], 256 patients should be recruited for an individual randomized study, to reach a variation coefficient of 40 mg/dl in total cholesterol levels, a difference of 15 mg/dl in the mean value of total cholesterol level among patients of the Intervention Group -IG- and the Control Group -CG-, and a deviation alpha = 0.05, beta = 15%.

As this is a cluster randomized study, "design effects" have been considered. The correlation intraclass coefficient estimates in cluster controlled clinical trials in primary care are generally below 0.05 [25]. For a cluster size of 15, this controlled clinical trial has a design effect of 1.7. Taking this value into consideration, the sample size should be 436 subjects (218 for each group).

### Participants

#### General Practices

We plan to recruit 48-50 randomized GPs trained to assist 8-10 patients each. The physicians recruited must work in a public primary care center in Spain and express commitment to keep their post during the field work period of the study.

#### Patients

The inclusion criteria are:

1) Patients of both genders, aged between 40 and 75 years.

2) Patients must be assisted by their family doctor.

3) Patients must be diagnosed with dyslipidemia in accordance with the following classification [26]:

• Defined hypercholesterolemia: total cholesterol >250 mg/dl (6.45 mmol/l) and triglycerides <200 mg/dl (2.26 mmol/l);

• Hypertriglyceridemia: total cholesterol <200 mg/dl (5.17 mmol/l) and triglycerides >200 mg/dl (2.26 mmol/l).

• Mixed hyperlipidemia: total cholesterol >200 mg/dl (5.17 mmol/l) and triglycerides >200 mg/dl (2.26 mmol/l).

4) No lipid-lowering drug therapy at the time of inclusion.

5) Capacity to provide informed consent.

The exclusion criteria are:

• Patients with pathologies that can produce secondary dyslipidemIa and need pharmacological therapy;

• Subjects with previous cardiovascular events or other chronic conditions as diabetes or severe Chronic obstructive pulmonary disease, cancer, serious liver alterations, chronic renal failure, at-risk or alcoholic drinkers, drug users;

• Patients on long-term sick leave;

• Pregnant or nursing women;

• Subjects unable to comply with the study procedures for their personal characteristics (cognitive level, altered psychological status, etc)

#### Randomisation

The participating practitioners will be allocated to each arm of the study by using the simple randomization method in a 1:1 ratio for IG and CG, using the software EPIDAT 3.0.

#### Study Intervention

Patients in the IG will be assisted by a GP who will use the MI approach, while patients in the CG will be assisted as usual (informative model). Both groups of physicians will base their therapy on the scientific evidence provided in current clinical practice guides on the treatment of dyslipidemia.

#### Intervention Group

IG practitioners will assist their patients using the MI method in compliance with clinical protocol recommendations.

In this study, a MI training program will be implemented following the models already established in scientific literature and in recognized works by international authors currently associated in the Motivational Interviewing Network of Trainers (MINT), which meets all international research and dissemination standards.

At present, MI has been agreed to include eight tasks GPs should be able to perform [21]:

1. General spirit (collaborative thinking, respect for patients' personal autonomy...);

2. Patient-centered counseling (this involves the comfortable practice of open-ended questions, affirmation, summaries, and particularly the skill of accurate empathy as described by Rogers [10]);

3. Ability to recognize patients' change talk or counter-change arguments.

4. Eliciting and strengthening patients' change talk;

5. Rolling with resistance and counter-change arguments;

6. Developing a reasonable change plans suitable for the patient.

7. Consolidating patient's commitment by helping them develop specific change attitudes.

8. Ability to combine MI with other intervention methods.

**Figure 2:**
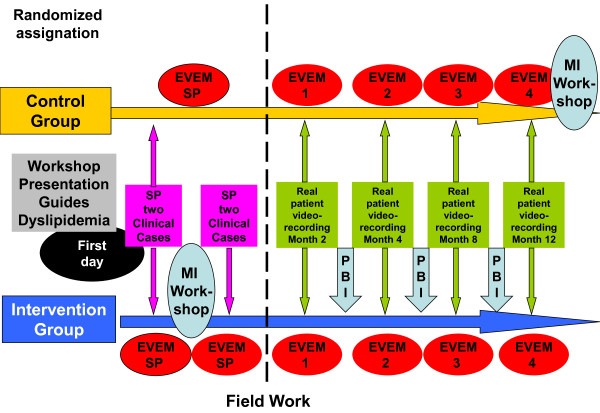
**Training program on Motivational Interview of Dislip-EM study**. MI: Motivational Interview; SP: Standard Patients; PBI: Problem Based Interview; EVEM: rating scale of motivational interviewing.

The training program on MI (Figure 2) will be implemented with the IG practitioners. The program includes classroom workshops and online training through a website-based training platform in Moodle format available at http://www.doctutor.es, where the tasks are developed and IG practitioners are monitored. The baseline Motivational Interviewing level of physicians is assessed by using the EVEM scale (Table 1) and a two-station clinical interview (OSCE model) with standardized patients and video recordings. Improvements in the level of MI skills will be assessed watching the video recordings at first visit and four visit. This study introduced an innovative learning technique which consists of watching the videotapes of the interviews uploaded on a virtual library with restricted access. Each participant can watch their interviews and assess their own position on the EVEM scale. Participants' self-assessment is contrasted by expert testers. Subsequently, through the platform or via e-mail, testers provide the practitioners with feedback for them to reflect on the results (motivational teaching method, in contrast with the directive method).

**Table 1:** EVEM scale version 1.3

ID code: Behavior studied: Time devoted to the interview (min):				
To what extend does the professional...	0	1	2	NA
1. tune with the patient through non-verbal communication?				
2. show empathy at appropriate times?				
3. make proper patient positioning concerning the behavior in question?				
4. works consistently with the positioning of the patient throughout the interview?				
5. use open-end questions?				
6. validates genuinely the patient (abilities, skills, effort, interest...) ?				
7. perform reflective listening?				
8. make summaries of the information provided by the patient?				
9. strengthens change talk?				
10. give attention to the patient's commitment to change?				
11. identifies resistance to change and use specific strategies to avoid and handle them?				
12. provides information adapted to the patient's difficulties and needs?				
13. promotes the definition and/or prioritization of the objectives of change with the patient?				
14. negotiates and test a feasible action plan considering the patient's options?				
15. once change has started, develop maintenance strategies with the patient?				
16. in case of relapse, create a climate of acceptance, trying to promote patient's self-efficiency?				

NA: Not Applicable

The sequential development of the MI training program consists of the following sections:

1. All practitioners -IG and CG- will attend a four-hour workshop where the study and clinical practice guides on dyslipidemia will be presented.

2. A standardized classroom workshop on MI will be held focusing on the eight internationally recognized stages (16 hours distributed in two consecutive days). The workshop is to be held in the three sites of the project with the same trainer. All workshops will be video recorded to verify that the eight stages were explained.

3. Video recording of two interviews with standardized patients (trained by the same trainers) before and immediately after the workshop on MI.

4. After watching the videotapes, feedback will be provided by a MI expert in person.

5. Creation of a video library in Vimeo http://www.vimeo.com with all the videotapes randomly numbered (combining all the sites).

6. "Educational micropills" will be forwarded to the participants via Internet and short messages during the months after the course (up to a year after the beginning of the course).

7. Interviews with the real dyslipidemic patients participating in the study (2-4-8-12-month visits) will be video recorded.

8. Task performance and feedback: critical incident, reading and commenting an article on Motivational Interviewing, watching their own video recordings uploaded on the platform, attendance to one or two Problem Based Interview (PBI) sessions [27] to watch and analyze a video with the IG researchers of their city.

9. At the end of the training period, a one-hour presentation on MI will be given by GPs to their team mates.

One of the most innovative aspects of the program is the use of modern online technologies:

1. Second life: The project was designed by a group of experts who live in different cities in Spain, and who make up the coordination team. This site allows researchers to hold regular meetings on-line. Follow-up will be performed through scheduled meetings using semFYC island software http://secondlife.com/.

2. Skype. Some of the training program coordinators will meet via skype. The option "share screen" will allow the team to prepare new documents in real time.

3. Moodle platform. Moodle is a Course Management System (CMS), also known as a Learning Management System (LMS) or a Virtual Learning Environment (VLE). It is a **f**ree web application that educators can use to create effective online learning sites http://www.moodle.org. The intervention group monitors the study using the Moodle platform, which contents are edited and integrated into the website Doctutor, thus allowing researchers consult all documents, materials and project guides, monitor the schedule, perform training tasks, interact via a blog, and receive feedback from their tutors (E-learning). Expert trainers/tutors can also use a platform for collaborative work.

4. E-mail and short messages. Each month, participants will receive "training micropills" on their e-mail address apmotivacional@gmail.com. These micropills are on the eight basic stages of MI training. They can also be accessed via Moddle.

- Videotape Analysis. It is performed using the EVEM scale (Table 1), which is currently being concurrently validated in the project itself. This scale will be used by GPs for self-assessment (self-perception) and by the researchers (peer review). The inter-observer concordance level will be analyzed.

#### Control Group

The GPs assigned to this group will provide the usual "brief counseling" based on medical advice, where patients will be warned about the need to change their habits into healthier ones. GPs will provide the recommendations included in the clinical protocols concerning diet, physical activity and tobacco consumption. The 16-hour workshop on motivational Interviewing will be also provided to the CG practitioners once the study is completed.

#### Outcome Measurement and Tools

At month 2, 4, 8 and 12, the results will be assessed using the following measuring instruments and procedures:

-Blood analysis: total cholesterol levels, LDL and HDL cholesterol, and triglycerides.

-Cardiovascular risk: to assess cardiovascular risk, we will use the SCORE table for low-risk European population [28,29], the table of the REGICOR study [30], and Framingham's equations [31] by using the application Circe.exe http://www.1aria.com/sections/cardiovascular/hipertension/HipertensionCalculadorasRiesgo.aspx that, when data are entered, automatically calculates the figures corresponding to each cardiovascular risk function.

-Anthropometric data: weight, height and body mass index (BMI: kg/m2).

-Overweight/Obesity: waist circumference measurement according to the criteria of the World Health Organization [32] and the amendments proposed by the Sociedad Española para el Estudio de la Obesidad (SEEDO) for Spain [33].

-Diet: questionnaire on adherence to the Mediterranean diet, validated for Spain [34].

-Physical activity and sedentary behavior: the International Physical Activity Questionnaire -IPAQ- [35] will be used to quantify the level of physical activity.

The primary analysis will be centered on the level of accomplishment of the recommended objectives in patients with dyslipidemia according to recommendations of the European Cardiovascular Prevention Guide, Spanish adaptation of 2008 [36]:

- Total cholesterol < 200 mg/dl (5.2 mmol/l) and LDL cholesterol <130 mg/dl (3.4 mmol/l).

- Severe hyperlipidemia total cholesterol ≥ 320 mg/dl(8 mmol/l) or LDL cholesterol ≥ 240 mg/dl (6 mmol/), considered high-risk patients: to reduce total cholesterol <175 mg/dl - <4.5 mmol/l- (155 mg/dL if possible) and LDL cholesterol <100 mg/dl - <2.5 mmol/l- (<80 mg/dL if possible).

### Trial Procedure

#### Recruitment

##### Practice recruitment

A number of practitioners were invited through different means (Sociedad Española de Medicina Familiar y Comunitaria, project coordinators' peers, scientific events, etc) to participate in the study. Those who were willing to participate (a total of 50 GPs from different regions in Spain) signed a commitment and confidentiality document.

##### Patient recruitment

Patients will be recruited using the case finding method; the procedure will consist on actively searching subjects who are eligible and invite them to participate in the study in a consecutive way, until the sample size planned is reached.

### Analysis

#### Primary Analysis

The primary objective of the study is to assess changes and differences in cholesterol levels and cardiovascular risk between both groups, in terms of whether the recommended therapeutic objectives were achieved or not.

Some of the secondary variables of the study are to take anthropometric measures, which will allow researchers assess changes in patients' weight and body composition (weight, height, BMI, waist circumference), as well as assessing their degree of adherence to the Mediterranean diet and evaluating changes in physical activity habits.

All intervention effectiveness analysis will be performed by intention to treat complying with the cluster design and considering two levels (practitioner and patient). The multi-level logistic regression analysis will be performed using MLwiN version 2.02.

#### Process Evaluation

The evaluation process will allow us to verify and monitor the impact of the different aspects of the intervention:

-Video recordings with standardized and real patients.

-Monthly contacts via telephone and at least one visit to the center of each of the participating researchers.

-Validation and use of EVEM for the evaluation.

-As a self-control and reinforcing mechanism, after the interview, professionals will fill in an autocheck-list by using the EVEM questionnaire (table 1).

## Discussion

This project represents an attempt to advance on an aspect of health care that is considered key in Western health systems today due to its high magnitude: testing the most effective preventive approaches in the fight against Heart Disease in primary care.

There are few publications that demonstrate the effectiveness of nonpharmacological interventions in the treatment of dyslipidemia. Demonstrating which of the two interventions tested here is more effective can have a major impact on clinical practice when designing and proposing clinical protocols for the prevention of CRFs.

Clinical Interviewing skills and health education are the essential strategy used by general practitioners with their patients. Obtaining scientific evidence on the effectiveness of MI in primary care through strict monitoring of the training methods, and controlling the integrity of the therapies applied, will allow us to have economical brief, effective and applicable techniques to help patients change their health habits and achieve better health outcomes.

This study is one of the first attempts to assess to what extent the motivational approach -as defined in MI (and their EVEM adaptation) is applied by GPs. The results obtained will help us understand the practical effectiveness of this approach, including its limitations and actual impact on cardiovascular health. If this method proved to be effective, our intention is to disseminate and promote that interventions based on the training model tested would be included into the clinical practice guide usually employed by family doctors.

### Protection Against Bias

The study is open, so the intervention can not be masked. Therefore both, patients and practitioners, will know who is involved in the intervention, and this could condition their response (Hawthorne effect), specially in the control group. Just the fact that the participants are identified and express their wish to participate in the study, and subjecting them to more intensive monitoring than usual, could make them more willing to follow medical advice.

To control and monitor the interventions, some of the clinical interviews will be video recorded, which may lead to a bias. To compensate this, our intention is to ensure that both groups differ only in the type of intervention, and to verify that only the physicians of the experimental group perform MI, while the control group does not use this approach.

Logically, the fact that doctors know that they are being observed may affect their performance and lead them to change their behavior, but there is wide experience in the use of observation methods similar to the one used here confirming that, in real practice, it is very difficult to modify the style of consultation used. In any case, if there is any bias, it will not be differential, since both groups are subject to the same observation procedure. Changes in patient behavior tend to be even lower [37].

The study will be randomized by cluster (professional) to avoid contamination bias and ensure recruitment of the adequate number of patients. We hope that randomization will allow to reach a balance between the two groups concerning the characteristics both of the physicians and the patients involved. Potential confounding factors are to be controlled by multivariate analysis.

## Competing interests

The authors declare that they have no competing interests.

## Authors' contributions

LAPT is the principal Investigator who conceived the study and led the study design and funding application. Contributed to the Statistical Analysis Plan. Led the writing of this manuscript.

JMB, JBF, NBB, MCN, JCA, JAP, FB, JM, RM, JAF, EMR, JRC contributed to the study design, funding application, study implementation and the intervention development. Contributed to writing the paper.

MP, MCL and IOC contributed to data collection and input.

All authors contributed to, read and approved the final version of the manuscript.

## Appendix

Participants in the Collaborative Group of the *Dislip-EM Study*

1. Emilio García Criado (CS Fuensanta. Córdoba. Spain)

2. María Pineda Alonso (CS Levante sur. Córdoba. Spain)

3. Ana Roldán Villalobos (CS Huerta de la Reina. Córdoba. Spain)

4. Antonio Pérez Fuentes (Consultorio Villafranca de Córdoba. Spain)

5. Mª José Acosta García (CS Adamuz. Spain)

6. Victoriano Rodríguez Navarro (CS La Carlota. Spain)

7. Isabel de Andrés Cara (CS Levante sur. Córdoba. Spain)

8. Antonio León Dugo (CS Levante sur. Córdoba. Spain)

9. Alfredo Ortiz Arjona (CS La Carlota. Spain)

10. Pilar Serrano Varo (CS Posadas. Spain)

11. Antonio Valero Martín (Consultorio Villafranca de Córdoba)

12. Juan Manuel Parras Rejano (CS Peñarroya. Spain)

13. Rosana Izquierdo Fernández (CS Coruxo. Spain)

14. José Antonio Pérez Vences (CS Rúa Cuba. Spain)

15. Antonio Fernández Crespo (CS Colmeiro. Spain)

16. Susana Hernaiz Valero (CS Val Miñor. Spain)

17. Mª Jesús Cobas Martínez (CS Matamá. Spain)

18. Neus Fernández Danés (ABS Centre L' Hospitalet de Llobregat. Spain)

19. Francisca Pérez Fuentes (CS Virgen Linarejos. Linares. Spain)

20. Clara Soria López (CS Virgen Concha. Zamora. Spain)

21. Juan Carlos Verdes-Montenegro Atalaya (CS Comuneros. Burgos. Spain)

22. Silvia Membrilla Pastor (CAP Ramona Vía.El Prat de Llobregat. Spain)

23. Francisco Mora Moreno (CS Molino de la Vega. Huelva. Spain)

24. José Luís Montero Monterroso (CS Fernán Núñez. Spain)

25. Mª Dolores Vargas Rubio (CS Fernán Núñez. Spain)

26. Antonio López Hernández (CS Posadas. Spain)

27. Santiago Avilés Cigüela (ABS Centre L' Hospitalet de Llobregat. Spain)

28. Susana Aldecoa Landesa (Centro Saúde Beiramar. Spain)

29. Félix Suárez González (CS San Roque. Badajoz. Spain)

30. Cristina Aguado Taberné (CS Santa Rosa. Córdoba. Spain)

31. Manuel Rico Cabrera (CS Villaviciosa de Córdoba. Spain)

32. Francisco Caro Tejero (CS Bujalance. Spain)

33. Silvia Díez Moreno (CS Tui. Pontevedra. Spain)

34. Gina Ballester Adell (CAP Vallcarca Sant Gervasi.Barcelona. Spain)

35. Alexis Tena Domingo (CAP Vallcarca Sant Gervasi. Barcelona. Spain)

36. Juantxo Mendive Arbeloa (CAP La Mina.S. Adriá de Besos. Spain)

37. Mª Dolores Pazo Ferreiro (CS Pintor Colmeiro. Vigo. Spain)

38. Azucena Carranzo Tomás (CAP Vallcarca Sant Gervasi.Barcelona. Spain)

39. Laura Belmonte Calderón (CS "El Castell".Castelldefels. Spain)

40. Miriam Ruíz Sánchez (ABS Centre L' Hospitalet de Llobregat. Spain)

41. Cristina Ortodó Parra(ABS Centre L' Hospitalet de Llobregat. Spain)

42. Sonia Cibrián Sánchez (CAP Vallcarca Sant Gervasi.Barcelona. Spain)

## Pre-publication history

The pre-publication history for this paper can be accessed here:

http://www.biomedcentral.com/1471-2296/12/125/prepub
